# Combination of robot-assisted laparoscopy and ureteroscopy for the management of complex ureteral strictures

**DOI:** 10.1186/s12894-023-01333-3

**Published:** 2023-10-12

**Authors:** Wenjin Yang, Weinan Tang, Xi Zheng, Mengjie Zhang, Xinyi Lu, Zeqing Chen, Changwei Ji, Hongqian Guo

**Affiliations:** 1 Department of Urology, Nanjing Drum Tower Hospital Clinical College of Nanjing University of Chinese Medicine, 321 Zhongshan Road, Nanjing, 210008 China; 2 Department of Urology, Nanjing Drum Tower Hospital Clinical College of Xuzhou Medical University, 321 Zhongshan Road, Nanjing, 210008 China; 3 Department of Urology, Nanjing Drum Tower Hospital, The Affiliated Hospital of Nanjing University Medical School, 321 Zhongshan Road, Nanjing, 210008 China

**Keywords:** Ureteral strictures, Robot-assisted surgery, Ureteroscopy, Surgical technique, End-to-end anastomosis

## Abstract

**Background:**

To summarize the efficacy of combined robot-assisted laparoscopy and ureteroscopy in treating complex ureteral strictures.

**Methods:**

Eleven patients underwent combined robot-assisted laparoscopy and ureteroscopy for ureteral strictures between January 2020 and August 2022. Preoperative B-ultrasound, glomerular filtration rate measurement, and intravenous pyelography showed different degrees of hydronephrosis in the affected kidney and moderate to severe stenosis in the corresponding part of the ureter. During the operation, stricture segment resection and end-to-end anastomosis were performed using the da Vinci robot to find the stricture point under the guidance of a ureteroscopic light source in the lateral or supine lithotomy position.

**Results:**

All the patients underwent robot-assisted laparoscopy and ureteroscopy combined with end-to-end ureterostenosis. There were no conversions to open surgery or intraoperative complications. Significant ureteral stricture segments were found in all patients intraoperatively; however, stricture length was not significantly different from the imaging findings. Patients were followed up for 3–27 months. Two months postoperatively, the double-J stent was removed, a ureteroscopy was performed, the ureteral mucosa at the end-to-end anastomosis grew well, and the lumen was patent in all patients. Furthermore, imaging examination showed that hydronephrosis was significantly improved in all patients, with grade I hydronephrosis in three cases and grade 0 hydronephrosis in eight cases. No recurrence of ureteral stricture was observed in patients followed up for > 1 year.

**Conclusion:**

Robot-assisted laparoscopy combined with ureteroscopy is an effective method for treating complex ureteral strictures and can achieve accurate localization of the structured segment.

## Background

The incidence of ureteral strictures has increased in recent years. Ureteral strictures are caused by many factors. In addition to congenital stricture, acquired surgery, especially interventional lithotripsy, has become the main cause of acquired stricture. Approximately 2.9–3% of patients present with stricture after ureteroscopy, 1.5% after shock wave lithotripsy, and 2.6% after shock wave lithotripsy combined with ureteroscopy [1, 2].

Patients who develop ureteral strictures may have varying degrees of hydronephrosis or renal obstruction, which may silently lead to progressive loss of ipsilateral renal function. Therefore, early and timely intervention is required when ureteral strictures are present [3]. Accurate timing and convenient treatment modalities are essential for maintaining renal function [4].

Currently, treatment methods for ureteral stricture include end-to-end ureteral anastomosis, ureteral balloon dilatation, ureteral stricture incision, Permanent ureteral stent implantation, and various types of mucosal replacement of the ureter [5]. End-to-end ureteral anastomosis is the most common treatment for ureteral strictures owing to its definite efficacy and low probability of postoperative restenosis. Midshaft ureteral strictures of < 2 cm in length are usually treated using end-to-end anastomosis [6]. However, generally, when the ureteral stricture segment is resected during surgery, it depends greatly on the surgeon’s subjective judgment, and errors in judgment often occur, resulting in incomplete resection of the ureteral stricture segment or misresection of the normal ureter. The present study used robot-assisted laparoscopy combined with ureteroscopy for end-to-end ureteral anastomosis. Notably, a ureteroscopy examination can accurately locate the narrow segment of the ureter. Furthermore, the light source of the ureteroscope can play a role in laparoscopy, guiding the surgeon in accurately removing the narrow segment under the robotic laparoscopic lens. In addition, the robotic platform is appropriate for ureteral reconstruction because it maintains the advantage of minimally invasive surgery and allows the surgeon to see in magnified three-dimensional vision, operate in limited space, and suture precisely. Robot-assisted ureteral reconstruction has been increasingly used because of its good outcomes [7]. We retrospectively reviewed the medical records of 11 patients who underwent combined robot-assisted laparoscopic and ureteroscopy for ureteral strictures at our center between January 2020 and August 2022. We described the surgical technique, summarized the surgical experience, and explored the efficacy and safety of this surgical approach in depth.

## Materials and methods

### Patient cohort

A total of 68 patients underwent surgery for ureteral strictures between January 2020 and August 2022. Of these, 11 underwent combined robot-assisted laparoscopy and ureteroscopy to treat ureteral strictures. In the remaining 57 patients, the dilated ureter and stenotic ring could be observed under direct vision, the stenotic segment had an obvious anatomical location on imaging, and the stenotic part could be accurately located during the operation; consequently, ureteroscopy was not performed during the operation. The indication for dual-lens combined therapy is stenosis observable on imaging but cannot be judged by the naked eye under the surgical field of view.

We used descriptive statistics to analyze patient demographics and perioperative outcomes. Before operation, we estimated the stenosis length by imaging methods such as radiography or computed tomography(CT).

### Surgical technique

#### Patient positioning and robot set-up

All procedures were performed using the da Vinci® Si or Xi Surgical System (Intuitive Surgery, Sunnyvale, CA, USA) and ureteroscopy. Peritoneal and transurethral routes were used. After general anesthesia, the surgical position was selected based on the preoperative assessment of the ureteral stricture location.

#### Positioning

**Fig. 1:**
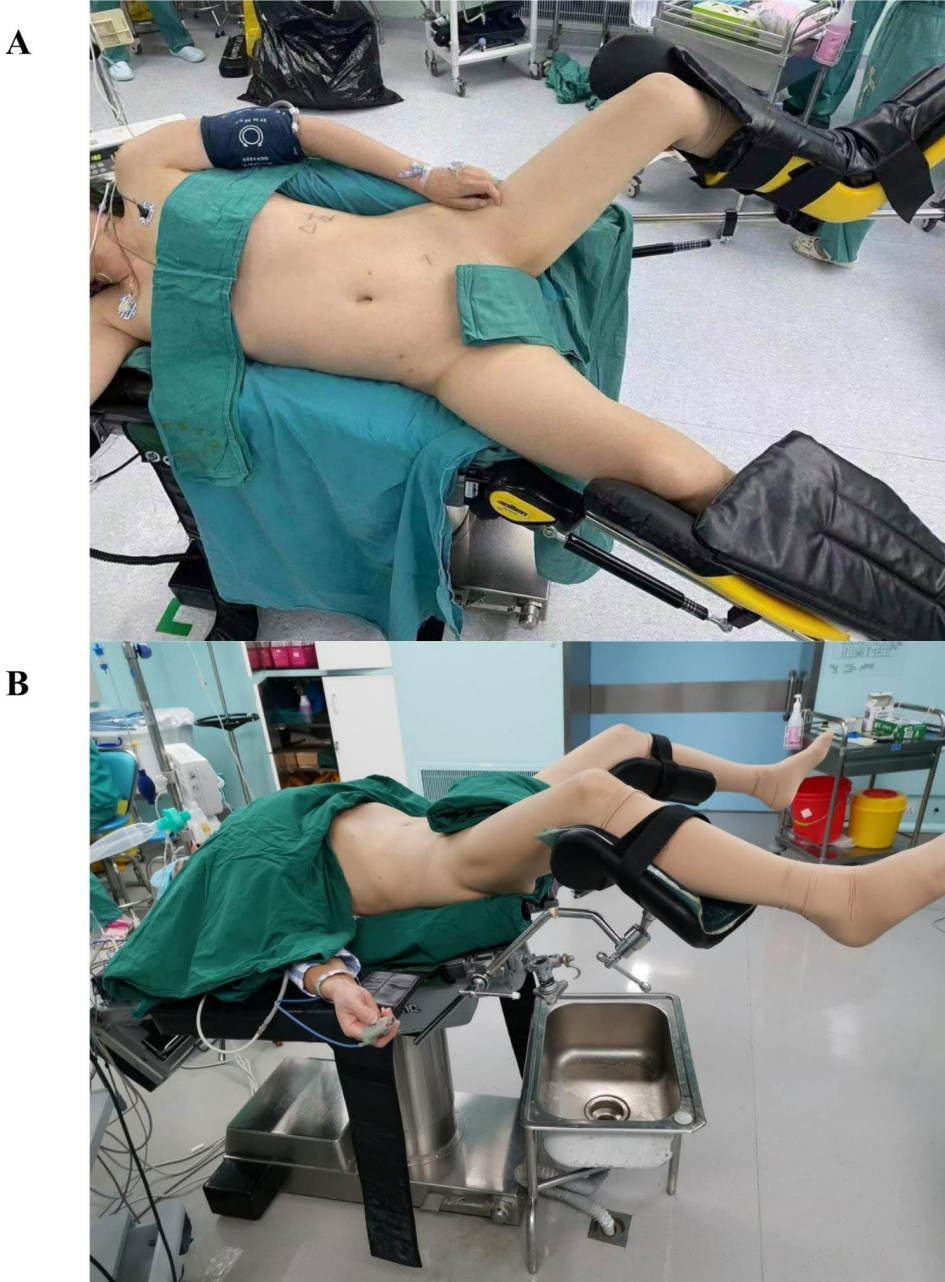
Patient positioning **A**: Lateral lithotomy position; **B**: Supine lithotomy position

When the stenosis was located above the crossing of the ureter and iliac vessels, a lateral lithotomy position was used (Fig. 1A), whereas when the stenosis was located below the crossing of the ureter and iliac vessels, a supine lithotomy position was used, with the head low and foot high (Fig. 1B).

#### Distribution of puncture cannula

**Fig. 2:**
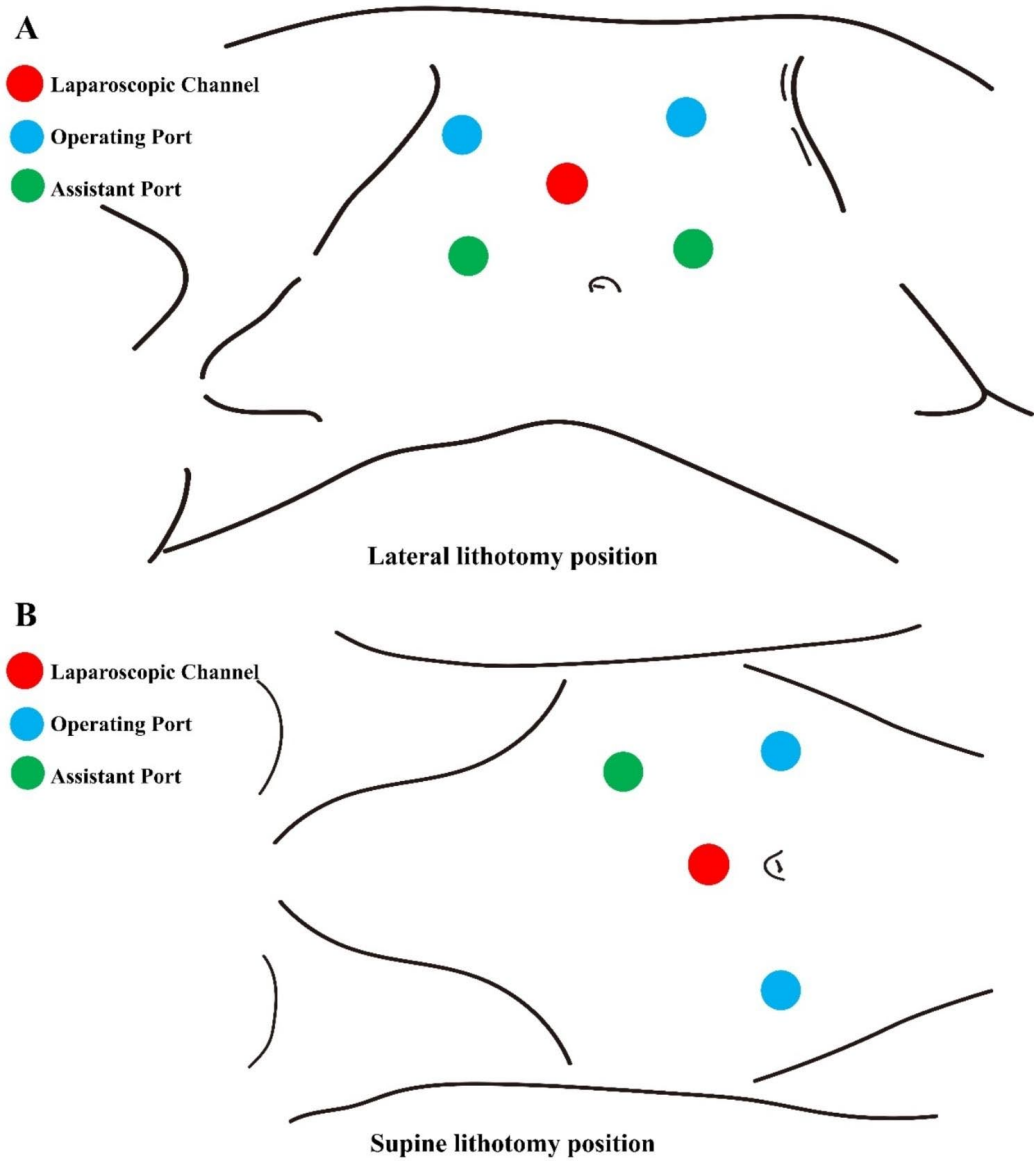
A Distribution of puncture cannula **A**: Lateral lithotomy position; **B**: Supine lithotomy position

1. Lateral lithotomy position (Fig. 2A): A paraumbilical approach was generally used. After pneumoperitoneum was established, a longitudinal incision of 10 mm was made at the lateral border of the rectus abdominis muscle of the two transverse fingers above the umbilicus, and a 12 mm puncture sheath was inserted as a laparoscopic channel. Another proximal 8 mm cannula was located in two transverse fingers below the costal margin of the midclavicular line, approximately 10 cm from the laparoscopic channel. The distal 8 mm cannula was placed at the midpoint of the line between the laparoscopic channel and anterior superior iliac spine. The assistant channel positioning was connected with three points of the laparoscopic channel and operation hole, forming an equilateral triangle, one on the left and another on the right. Furthermore, a 5-mm cannula was placed under the xiphoid process during right ureteral surgery to elevate the liver.

2. Supine lithotomy position (Fig. 2B): After pneumoperitoneum was established, a longitudinal incision of 10 mm was made in the transverse finger just above the umbilicus, and a 12 mm puncture sheath was inserted as a laparoscopic channel. Operating ports on both sides were selected at the level of the flat umbilicus, approximately 8–10 cm from the umbilicus. The assistant port was selected approximately 10 cm above the lateral aspect of the left robotic arm to form an equilateral triangle with the laparoscopic channel and left operating port.

### Release and ureter incision, narrow part localization

#### Ureteral separation

The surgeon used the da Vinci surgical robot to free the ureter from the surrounding tissue.

**Middle and upper ureteral separation**: The lateral peritoneum was opened along the paracolic ligament, the hepatic (splenic) colonic ligament was divided, and the ascending (descending) colon was turned inward and downward to fully expose the middle and lower poles of the kidney, respectively. The perirenal fascia and surface tissue of the renal pelvis were opened. The renal pelvis was fully freed, and the middle and upper ureters were freed by separating them inferiorly along the renal pelvis. This frees and protects the blood supply to the ureter.

**Middle and lower ureteral separation**: The lateral peritoneum was opened from the external iliac artery to locate the ureter across the external iliac artery, and the middle and lower ureters were separated superiorly and inferiorly along the ureter.

#### Stricture location

**Fig. 3:**
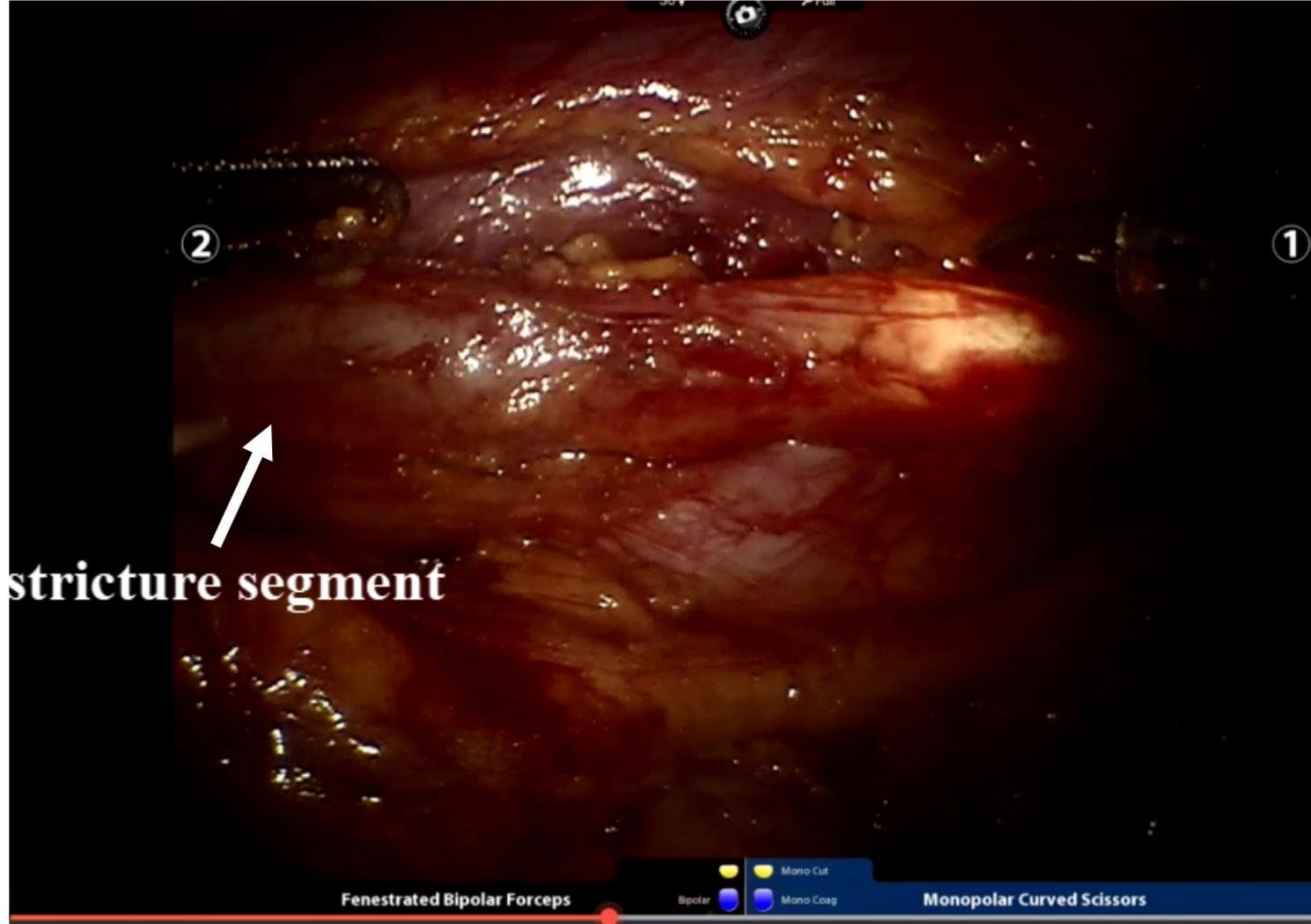
Intraoperative picture

While the chief surgeon was operating the da Vinci surgical robot, another group of surgical team members was preparing for ureteroscopy beside the operating table. As the chief surgeon was about to complete dissociation of the ureter, a ureteroscope was inserted through the urethra into the affected ureter using a guidewire. A significant stricture ring was observed, and the guidewire could not pass through. The luminance of the laparoscopic light source was lowered, guided by ureteroscopic light, which guided the surgeon to divide the distal end of the narrow segment. (Fig. 3) Urine outflow from the proximal ureter was observed. Under the direct vision of the robot lens hole, the proximal ureteral stenosis was stripped until the guidewire could pass smoothly. One F6 double-J tube was placed over the guidewire.

#### End-to-end anastomosis

One centimeter each of the proximal and distal ends of the transected ureter were longitudinally dissected, and tension-free anastomosis was performed using robot-assisted laparoscopic surgical procedures. Abdominal drains were left in place after checking for the absence of active bleeding, and the procedure was completed by suturing the incisions on each side.

### Postoperative management and follow‑up

Patients were routinely administered symptomatic and supportive treatment such as antibiotics, analgesics, and fluid replacement postoperatively. On postoperative day 2, an abdominal radiograph was performed to check if the double-J tube was in place. After the postoperative drainage volume reached < 100 mL, the drainage tube was removed. The urinary catheter was removed 2–3 weeks postoperatively, and the double-J catheter was removed under a ureteroscope 2 months postoperatively. All patients underwent ureteroscopy to observe wound healing after end-to-end ureteral anastomosis. Three months after double-J catheter removal, computed tomography urography (CTU) was repeated.

Consequently, patients were reviewed every 6 months. The criteria for a successful operation was good anastomotic healing observed using the ureteroscope. Imaging examination showed that hydronephrosis on the affected side had significantly reduced or disappeared.

## Results

Of the 11 patients, six were males and five were females. Their age range was 29–56 years (median, 39 years). Eight patients had a history of transurethral ureteroscopic ureteral laser lithotripsy before stricture (no evidence of ureteral stricture before lithotripsy), whereas the remaining three had strictures of unknown cause. All patients underwent preoperative CTU examinations to confirm the diagnosis of ureteral stricture and different degrees of hydronephrosis. We graded the degree of hydronephrosis using Onen’s Alternative Grading System [8, 9]. A stricture segment < 2 cm met the surgical indications for this operation. Regarding the stricture site, there were eight left-sided and three right-sided cases. Regarding the stricture location, it was located in the upper ureter in three cases, the middle ureter in seven cases, and the lower ureter in one case. Stenosis length was 0.6–1.8 cm (median 1.1 cm) [Table 1].

**Table 1:** Patients’ information

No. Pts	Gender	Age(y)	BMI (kg/m2)	Stricture length (cm)	Procedure Time (min)	Bleeding Volume (ml)	Postoperative hospital stay(d)	Preoperative hydronephrosis grade	Postoperative Hydronephrosis grade	Remove catheter(d)	Mos Follow up	Clinical Success	Radiographic success
1	Male	50	21.97	1.4	170	20	4	III	0	19	27	Yes	Yes
2	Male	52	27.38	1.8	120	15	5	IV	I	29^*^	24	Yes	Yes
3	Male	37	24.21	1.4	190	100	7	III	I	22	22	Yes	Yes
4	Male	29	23.04	1.5	145	20	3	III	0	15	19	Yes	Yes
5	Female	43	18.51	1.3	140	50	4	III	0	20	16	Yes	Yes
6	Female	37	22.19	1.1	180	50	2	III	0	12	15	Yes	Yes
7	Male	56	23.34	1	195	120	5	III	0	18	14	Yes	Yes
8	Female	30	19.88	0.8	160	100	4	II	0	16	12	Yes	Yes
9	Female	39	20.31	0.6	150	50	4	III	0	23	10	Yes	Yes
10	Male	37	24.66	1.5	105	20	7	IV	I	21	6	Yes	Yes
11	Female	52	19.72	0.7	115	20	3	III	0	16	3	Yes	Yes
Median (rang)		39 (29–56)	22.19 (18.51- 27.38)	1.3 (0.6–1.8)	150 (105–195)	50 (15–120)	4 (2–7)			19 (15–29)	15 (3–27)		

*The patient had an underlying disease (prostatic hyperplasia), and 14 days postoperatively, dysuria and urinary retention occurred after the catheter was pulled out. To avoid disturbing the effect of the operation, a catheter was inserted again. After benign prostatic hyperplasia was treated with drugs, the catheter was removed 29 days operatively, and the patient could urinate on his own

In the remaining 57 patients, the dilated ureter and stenotic ring could be observed under direct vision or the stenotic segment had an obvious anatomical location on imaging, and the stenotic part could be accurately located during the operation. Consequently, ureteroscopy was not performed during the operation.

All the patients underwent robot-assisted laparoscopy and ureteroscopy combined with end-to-end ureterostenosis anastomosis. There were no conversions to open surgery or intraoperative complications. Significant ureteral stricture segments were found in all patients intraoperatively; moreover, stricture length was not significantly different from the imaging findings. Operation time was 105–195 min (median, 150 min), and intraoperative bleeding was 10–120 ml (median, 50 ml). Postoperative drain removal time and length of hospital stay were 1.8–2.8 days (median, 2.1 days), and 2–7 days (median, 4 days), respectively. Postoperative urinary catheter removal time was 12–29 days (median, 19 days). Postoperatively, two patients had a fever; however, their body temperature returned to normal after antibiotic treatment. Patients were followed up for 3–27 months (median, 15 months). Abdominal radiography was repeated 1–2 days postoperatively, and a double-J tube was placed in all patients. Two months postoperatively, the double-J catheter was removed, ureteroscopy was performed, the ureteral mucosa at the end-to-end anastomosis grew well, and the lumen was patent in all patients. Furthermore, imaging examination showed that hydronephrosis was significantly improved in all patients, with grade I hydronephrosis in three cases and grade 0 hydronephrosis in eight cases. No recurrence of ureteral stricture was observed in patients followed up for > 1 year.

## Discussion

In recent years, ureteral stones have become more common. With the development of ureteroscopy, a series of interventional stone diagnosis and treatment methods, such as ureteral laser lithotripsy, is becoming popular. The most common iatrogenic complication after laser lithotripsy is a secondary ureteral stricture [10]. The reason for this may be multifactorial [1]. In addition, the more prevalent use of high-power lasers increases energy, which directly affects the ureteral wall, and also increases the fluid temperature, which has been shown in various models to cause tissue damage and may lead to ureteral stricture disease [11, 12]. Inflammatory processes after injury produce fibrillar exudates that precipitate in traumatized areas, thereby promoting adhesion to produce chronic granulomas and, ultimately, strictures [13]. One of the most worrisome consequences of ureteral stricture formation is the loss of renal function due to silent obstruction [2].

Therefore, when a ureteral stricture is identified, it should be treated promptly. In the present study, we aimed to select from existing treatment options a more ideal and suitable regimen various ureteral strictures while reducing postoperative complications and the restenosis rate.

Currently, there are many ways to treat ureteral strictures. In the past, open surgery was often used to treat ureteral strictures. However, open surgery has been gradually replaced by minimally invasive surgery because of disadvantages such as long operation time, large blood loss, and many postoperative complications. Laparoscopic or minimally invasive surgery, as well as the combination of robot-assisted laparoscopy and ureteroscopy, is preferred to open surgery due to its advantages [14]. Common ureteral metal stent placement is not indicated for patients with severe pinpoint ureteral strictures. Daily activities may cause stent dislocation and prolapse, complicated by urinary tract infection and obstruction [15]. Therefore, ureteral stents are generally used after various stone surgeries, short-term temporary obstruction treatment, or very few cases that cannot tolerate major surgery. Ureteral balloon dilatation and ureteral stricture endopyelotomy are prone to excessive mucosal tears or perforations during surgery, resulting in urine leakage, hematuria, and other conditions. Furthermore, various studies have shown that the success rate of these two surgical methods is low [16, 17]. Mucosal replacement of the ureter is more suitable for patients with strictures of > 2 cm and high ureteral tension after resection of the ureter stricture, in whom end-to-end ureteral anastomosis cannot be performed. It is more appropriate to replace the ureter with the mucosa of autologous tissue [5, 18].

Therefore, end-to-end ureteral anastomosis is more appropriate for patients with strictures of < 2 cm, and the ureteral tension remains within the appropriate range after resection [5, 6]. End-to-end ureteral anastomosis can maximize the remodeling of ureteral morphology, maintain ureteral function, and reduce the risk of urinary system infection. Furthermore, because end-to-end anastomosis is an autologous tissue anastomosis, the postoperative ureteral mucosa recovers faster, and the probability of tissue rejection is smaller.

In the last few years, there have been rapid and continuously evolving technological developments in the surgical field. One of the most revolutionizing breakthroughs was the introduction of the Internet of Things(IoT) concept within the surgical practice [19]. The da Vinci robot operating system used in our surgery is one of the typical representatives of the Internet of Surgical Things. The superimposed real-time image guidance provided by the Internet of Things can provide surgeons with audio-visual guidance to observe the operation site and facilitate the implementation of the operation. It is important achieve the goal of faster suturing and less bleeding during the operation, which is necessary for postoperative recovery of patients and reduces the occurrence of perioperative complications [18].

Conventional laparoscopic or robot-assisted laparoscopic surgery for ureteral stricture resection and end-to-end anastomosis have limitations, such as a large amount of fibrous exudation around the ureter of the stricture segment, inaccurate localization of the ureteral stricture segment, and blindness. Consequently, when double-J stenting is performed, it is not guided and can easily cause secondary injury to the ureteral wall. Furthermore, many international researchers have performed specific staining or fluorescent agent localization techniques to address localization problems. This approach presents difficulties in surgical implementation in patients with a history of retroperitoneal fibrosis, previous abdominal or pelvic surgery, and radiotherapy. It is difficult to identify the narrow segment of the ureter because the underlying pathological factors lead to the disappearance of normal fascial planes or ureteral fibrosis encasement. In the absence of tactile feedback, fluorophore or stain localization relies primarily on visual cues and is not as intuitively observed as under direct ureteral visualization, which may lead to blindness, and iatrogenic injury may lead to devastating complications [20]. Furthermore, some reagents are expensive, and the operation is more complex, making it difficult to perform in China. Therefore, end-to-end anastomosis of the ureteral stricture segment is more appropriate for ureteroscopic light-source guidance. Using a ureteroscope, the stenosis can be reached under direct vision, and the guidance of the light source makes the resection of the stenosis more precise, avoiding the blindness of manual subjective operation [21]. At the same time, ureteroscopy is an advanced technology, and the preoperative positioning and addition of ureteral examination steps during the operation will not prolong the operation time. The direct vision guidance of the ureteroscope light can be said to be minimal effort that produces huge benefits [22].

When laparoscopy and ureteroscopy are combined with end-to-end ureteral anastomosis, the problem of positioning the ureteral stricture segment can be solved. However, common laparoscopic surgery often takes longer to separate the stricture segment than robotic laparoscopic surgery. In patients who experience severe adhesion between the ureter and surrounding tissues during complex ureteral stricture surgery, laparoscopic separation and deployment are often difficult to separate quickly. Furthermore, the robotic arm can achieve control of the ureteral clamp force, reduce unnecessary clamping and excessive force, and avoid the problem of postoperative ureteral edema. Under light-source guidance, precise resection of the stenotic segment is possible. When performing end-to-end anastomosis, the robot has faster suture speed and more precise anastomosis docking, which can effectively reduce intraoperative urine leakage and promote postoperative ureteral recovery [7]. Patients benefit from this minimally invasive approach, which reduces blood loss, hospital stay, and postoperative pain. Robot-assisted laparoscopic distal ureteral reconstruction has shown efficacy and feasibility in contemporary urological practice in appropriately selected patients [23].

The present study retrospectively analyzed 11 patients who underwent laparoscopy combined with ureteral stricture treatment at our center between January 2020 and August 2022. All the patients underwent open surgery without infection or postoperative urine leakage. During postoperative follow-up, ureteroscopy showed good mucosal growth of the ureteral wall and smooth lumen, and imaging examination showed that hydronephrosis was significantly reduced. No ureteral stricture was found in any patients who were followed up for 1 year postoperatively. The proposed treatment was safe and effective in all patients.

The results showed the safety and efficacy of the proposed treatment procedure; however, our surgical approach had some limitations. First, end-to-end anastomosis could not be used to treat patients with ureteral strictures of > 2 cm. Second, at a later stage, it is necessary to explore the method of accurate intraoperative localization, safety, and efficacy of robot-assisted laparoscopy combined with ureteroscopy in the treatment of long ureteral stricture segments for mucosal replacement plastic.

### Surgical key

1. The proximal and distal ends of the ureteral stricture segment must be fully freed, and the lumbar bridge may not be pulled in the lateral decubitus position to avoid hyperextension of the lumbar region and ensure a tension-free anastomosis.
2. Preserving the surrounding fascial layer and protecting the ureteral blood supply is essential during ureteral mobilization.
3. A posterior peritoneal reconstruction should be performed after ureteral anastomosis to restore the retroperitoneal anatomy of the ureter.

## Conclusion

Robot-assisted laparoscopy combined with ureteroscopy is an effective method for treating complex ureteral strictures and can achieve accurate localization of the structured segment. The localization method is simple and easy to perform in China. Further studies with larger cohorts and longer follow-up periods are required to clarify the role of robot-assisted laparoscopy in *managing* complex ureteral strictures.

## Data Availability

All data generated or analyzed during this study are included in this published article [and its supplementary information files].

## Abbreviations

CT: Computed Tomography
CTU: Computed Tomography Urography
IoT: Internet of Things
